# Genetic variation, heritability and genotype by environment interaction of morphological traits in a tetraploid rose population

**DOI:** 10.1186/s12863-014-0146-z

**Published:** 2014-12-20

**Authors:** Virginia W Gitonga, Carole FS Koning-Boucoiran, Kathryn Verlinden, Oene Dolstra, Richard GF Visser, Chris Maliepaard, Frans A Krens

**Affiliations:** Wageningen UR Plant Breeding, Wageningen University and Research Centre, PO Box 386, 6700 AJ Wageningen, the Netherlands; Present address: Lex + East Africa, PO Box 1739, Naivasha, 20117 Kenya; Present address: Syngenta Seeds B.V, PO Box 2, 1600 AA Enkhuizen, the Netherlands

**Keywords:** Rose, Tetraploid, Genetics, Morphological traits, Heritability, Correlations, Genotype x environment

## Abstract

**Background:**

Global trade has ensured that the ornamental horticulture continues to grow worldwide, with rose hybrids being the most economically important genus (*Rosa x hybrida*). Due to changes in global trade and an increase in energy costs the ornamental industry has seen a shift in the production and sale of flowers from the US and Europe alone to production in Africa and Latin America. As Kenya is a major exporter of roses to Europe we studied the genetic variation and heritability of specific morphological traits in a tetraploid population grown in the Netherlands and in Kenya. The aim was to estimate genotype by environment interaction (G × E) and to investigate the implications of (G × E) for rose breeding.

**Results:**

A tetraploid rose population (K5) from a cross between two tetraploid parents was field tested over two seasons in the Netherlands (summer and winter) and two locations in Kenya (Nairobi and Njoro). Ten traits were compared per genotype across the four environments. There were differences in trait association across the four environments showing that the traits were partially influenced by the environment.

The traits that had a low ratio of σ^2^_ge_/σ^2^_g_ also showed a high value for heritability. For the traits number of petals, prickles on petioles, prickles on stems the interaction is minimal. For the traits chlorophyll content, stem width and side shoots we observed a much higher interaction ratio of 0.83, 1.43 and 3.13 respectively. The trait number of petals had the highest heritability of 0.96 and the lowest σ^2^_ge_/σ^2^_g_ ratio (0.08). The trait number of side shoots (SS) with the lowest heritability (0.40) also had the highest σ^2^_ge_/σ^2^_g_ ratio of 3.13.

**Conclusion:**

Attained by this experiment showed that we have different magnitudes of non-crossover G × E interactions. For the traits number of petals, prickles on stems and prickles on petioles with a low interaction and high heritability, selection can be done at any of the environments. Thus, these traits can be confirmed at the breeding site. For the traits stem width, side shoots and chlorophyll content that had a higher interaction selection for or against these traits should be done at the production location or at least be verified there.

**Electronic supplementary material:**

The online version of this article (doi:10.1186/s12863-014-0146-z) contains supplementary material, which is available to authorized users.

## Background

Cut roses have an estimated turnover of 768 million Euro in the Netherlands, compared to 294 million of the number two cut ornamental, chrysanthemum [1]. This makes *Rosa* the most important genus, economically, of ornamental horticulture. In the period 2000–2008, rose imports grew by 60% from 2.3 billion in 2000 to 3.7 billion in 2008 [2]. The area of cut rose production worldwide is expanding with a remarkable progress in the developing countries. The production area in the Netherlands has decreased from 932 hectares in 2000 to 532 hectares in 2009 while the production area in Africa has increased from 810 hectares in 1997 to an estimated 5000 hectares of which 2200 hectares are in Kenya. Higher elevation areas in the tropics are ideal for roses, as the daytime temperatures are moderate while the nights are relatively cold. At same time the amount of light during the day is very high.

Because of these trends, there are now two types of growers; the European growers found in the country where the breeding centre and market are located and the foreign growers, usually located in tropical countries, who export their flowers to Europe and the USA. Both growers are interested in high yields, vase life, disease resistance and novel colours. European growers aim for novel colours, big heads and longer stems. Due to the high production costs in Europe, novelties in the roses ask for a higher price at the flower auction to make production economically viable. With the increase in production in the tropics, postharvest longevity is becoming increasingly important [3], as there are more days between harvest and arrival on the market. Disease resistance is important because it lowers the cost of chemicals, reduces pollution of the production environment and ensures that the flowers are disease free upon arrival. The rise and fall of temperatures during transport has been found to be conducive for opportunistic diseases like botrytis. Prickle free stems are increasingly important due to the ease of handling and transport and low stem weight reducing the freight costs. Whilst traditional breeding objectives in Europe included higher productivity under lower temperatures, postharvest vase life and tolerance to pests and diseases [4,5], the favourable environmental conditions in the tropics mean that breeders no longer need to breed for lower temperature varieties and can concentrate on the improvement of other morphological traits.

Most recurrent blooming roses flower within several weeks of germination allowing selection for floral traits to be made relatively early compared to most woody species [6]. This allows breeders to remove all genotypes that are considered undesirable in a process called roughing. The high cost of greenhouses means that most commercial breeding programs typically rough out 75-95% of their seedlings at first bloom [7]. This in effect favours the selection of floral traits compared to other morphological traits that need a longer period to be fully evaluated because the population size in which this can be done is already reduced to 25%.

A good plant breeding program has to take into consideration the influence of the genotype by environment interactions and the correlations of important traits. This is because the growing areas are now located worldwide while the breeding is still done in temperate regions. There is limited information available about the stability and expression of most of the cut rose morphological traits as well as the correlation of the different traits. In order to improve the efficiency of breeding for quality traits in roses, understanding the variation of these traits in different environments is necessary. The main purpose of multi-environment trials is to observe stability of genotypes across the environments, the identification of superior genotypes and of the location that best represents the target environment for production. Rose growers believe that high altitudes and cool climates lead to deeper colours and longer stems. The lower altitudes give faster maturing, more vigorous plants leading to higher production. So far, a few genetic studies have been performed on a limited number of traits in diploid roses [8-10] and in tetraploid roses [6,11]. The objectives of this study were to evaluate the genetic variation and heritability of ten specific morphological traits, important for grower, transporter or consumer within a tetraploid rose population, to monitor the effect of different environments and to estimate genotype by environment (GxE) interactions, and to investigate the implications of G × E for rose breeding. We used a tetraploid mapping population which has been bred in the Netherlands and then planted in the Netherlands and a tropical country (Kenya), which is now the industry standard. This allows us to study the phenotypic traits in the transition from juvenility to full commercial production in the Netherlands and in Kenya as well as the genotype by environment interactions.

## Methods

### Plant material and environments

The K5 tetraploid rose population used in this study was described by Yan et al. [12] and Koning-Boucoiran et al. [6]. This population, which originally was comprised of 184 genotypes, is a result of a cross between the two tetraploid genotypes, P540 and P867. These parents were selected because they showed segregation for powdery mildew resistance, flower colour and presence and absence of prickles on stem and leaves.

Trials were established at three locations, The Netherlands, Wageningen**(**51°59'0"N, 5°40'0"E, 11 m altitude), Kenya, Nairobi**(**1°21'0"S, 36°43'0"E, 1833 m altitude), and Kenya, Njoro **(**0°17'0"S, 35°54'0"E, 2161 m altitude). In the Netherlands observations were made during the summer of 2007 and the winter 2007/2008. In Kenya the observations were made between January and July of 2009.

Rooted nodal cuttings of each genotype, including the parents, were produced by Terra Nigra B.V., a Dutch company that is active in the breeding, propagation and marketing of roses. In the Netherlands, these cuttings were planted in pairs in pots of coco peat in a greenhouse. The greenhouse was artificially lit to ensure a day length of 18 hrs, the temperature was kept at 20°C (day temperature) and 17°C (night temperature), and the relative humidity (RH) was kept between 80 and 90%. A randomized complete block design was set up with one replicate pot per block. Each pot had two plants of the same genotype. Cuttings were shipped to Kenya in 2009 and rooted at the Terra Nigra site in Naivasha (Kenya) before transplanting at the two sites in Kenya: Nairobi (Winchester farm) and Njoro (Agriflora farm). The plants were grown in soil with a spacing of 15 cm. The growers were instructed to follow accepted production practices (fertilization, pest control, watering, bending, disbudding and de-suckering) for rose production. The set up was an incomplete block as the process of producing cuttings in the Netherlands, rooting them in Naivasha and then transplanting to Nairobi and Njoro meant that some genotypes of the original 184 present in Wageningen, did not survive. In total, 148 genotypes were fully represented at the two locations in Kenya, as well as in Wageningen. In each location there were 2 plants per genotype. Four stems were selected in each plant and measurements of the various traits were taken. Thus in total per genotype per flush there were 8 measurements taken. These measurements were done twice, with each repetition described as a flush. The traits were measured at three locations; in the Netherlands in Wageningen (WAG) in 2007, and in Kenya in Nairobi (WIN) 2009 and in Njoro (AGR) 2009. In each location we measured two flushes. In Wageningen, the first and the second flush corresponded to summer (WAG-S) and winter (WAG-W) measurements respectively.

Preliminary analysis of the data using Genstat 16 [13] was conducted per location using flush × genotype as fixed factors and repeated with flush and genotype as random effects to confirm if there were any significant differences between the flushes. We observed significant differences between the WAG-S and WAG-W measurements and no significant differences between the two flushes in WIN and AGR (α = 0.001). As a consequence of this, the three locations henceforth were treated as four environments.

### Evaluation of phenotypic traits

**Figure 1:**
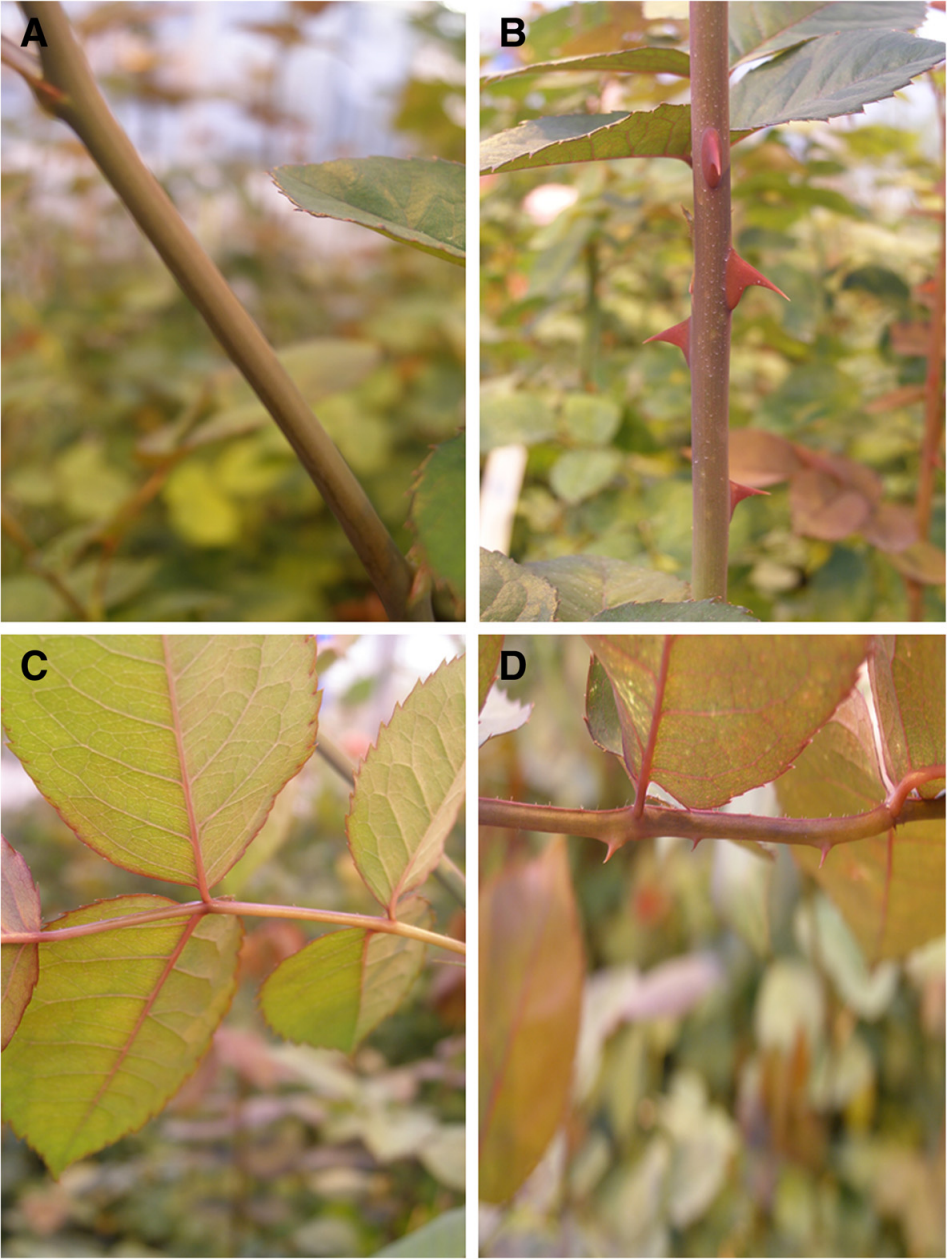
**The traits PS (prickles on stems, panels A and B) and PP (prickles on petioles, panels C & D) are shown here with examples of their range in the K5 progeny.** Panel **A** shows an individual without prickles on the stem and panel **B** with prickles on the stem. Panel **C** shows an individual without prickles on the petiole and panel **D** with.

In this study a number of horticulturally important traits were assessed. Three phenotypic growth traits were measured before the plants were bent. The bending of juvenile stems is a standard practice carried out before the plants can begin producing commercially viable stems. The growth traits were: plant Height (H), which was a measure of the height (cm) from the rim of the pot to the apical bud before bending; plant Vigour (V) where the plants were ranked on a scale of 1–5 based on their height, number of stems, number of leaves and the branching present at time of scoring; Bending date (BT) where the bending dates were given the following numerical scores: 1 = 29, 2 = 32, 3 = 37, 4 = 39, and 5 = 44 days after planting. These traits were only measured in Wageningen over summer (2007).The traits stem length (SL) which was the length (cm) from the floral tube to the shoot base, prickles on the stem (PS) which were the number of prickles between the 4th and 6th nodes on the main stem (Figure 1A and 1B), prickles on the petioles (PP) which were the number of prickles on the petioles (Figure 1C and 1D) that are formed between the 4th and 6th nodes and number of petals (NP) which were the number of petals counted when the flower was in full bloom, were measured in all environments at least 2 replicates of 4 individuals per genotype. The traits stem width (SW) which was the diameter (mm) of the stem at middle of the 2nd and 3rd internodes from shoot base, chlorophyll content (CHL) which was the chlorophyll content (mg/l) of the first fully-formed leaf from the top, using a portable fluorimeter (PAM-2001) Walz, Effeltich Germany, and side shoots (SS) which were the number of side shoots on the whole stem, were measured in Wageningen in winter (WAG-W) and in both flushes of WIN and AGR, for logistical reasons these traits were not measured in Wageningen in summer (WAG-S).

Temperatures and humidity measurements were taken in the greenhouse in all the locations [see Additional file 1].

### Data analysis

The traits were compared per genotype across the four environments. In order to estimate variance components of traits, a complete random linear model was used in the analysis of all the traits within and across the environments using the REML (Residual Maximum Likelihood) algorithm of Genstat 16 [13]. Descriptive statistics per environment and trait were obtained using the summary statistics procedure in Genstat 16 [13]. For each of the traits, an analysis of variance (ANOVA) was performed to estimate the means of the genotypes, the phenotypic and genetic variances, and the heritability.

### Heritabilities

The broad-sense heritabilities were calculated, across the environments and in each environment, using the following equation:

$$ {H}^2=\left({s_g}^2\right)/\left[{s_g}^2+\left({s_e}^2/r\right)\right] $$

Here s^2^_g_ and s^2^_e_ represent genetic variance and residual variance for each environment respectively and r the number of replicates of each genotype.

### Correlation analysis

Pearson correlation coefficients between the phenotypic traits were calculated per environment and over the environments to give a measure of the strength of linear association using Genstat 16 [13].

### Multivariate analyses

The trait data was graphically analysed across the four environments using principal components analysis (PCA). This was conducted using mean values from REML analysis of each trait using Genstat 16 [13]. PCA biplots enabled assessment of the genotypic variation on a multivariate scale, and the association among traits.

### Genotype by environment interaction

In order to quantify the size of the variation due to genotype × environment interaction relative to main genotypic variation, the morphological traits over locations were analysed using a linear mixed model with the residual maximum likelihood (REML) procedure of Genstat 16 [13]. In the linear mixed model the genotypes and genotype environment interaction were considered as random effects and the environments as fixed effects. Because we ultimately considered four environments, only 3 degrees of freedom were available for this term. We used the results of this mixed model to quantify, for the K5 population, the size of the genotype by environment interaction relative to the genetic variance using the ratio ${\sigma}_{ge}^2/{\sigma}_g^2$.

The genotype and genotype × environment (GGE) biplot was used to explain the variation due to genotypes and genotype × environments (G × E). The GGE biplot analysis was done using Genstat 16 [13].

## Results

### Greenhouse environment

The highest average relative humidity was observed in WAG-W at 95% followed by WAG-S at 82%. The average relative humidity in Kenya was considerably lower at 58% in WIN and 49% in AGR. The lowest average temperature was recorded in WAG-W at 19°C followed by WAG-S at 21°C. The average temperature in Kenya was recorded at 28°C in WIN and 25°C in AGR. WAG-W also had the lowest range of temperature with difference of 6°C compared to WAG-S that had a difference of 24°C [see Additional file 1].

### Phenotypic data

In Wageningen, The Netherlands there was significant genotype × flush interaction (*p <* 0.001) for stem length (SL) and number of petals (NP). Number of side shoots (SS), stem width (SW) and chlorophyll content (CHL) were only measured in the second flush. For these two reasons we opted to analyse Wageningen flush 1 and flush 2 as two different environments, Wageningen summer (WAG-S) and Wageningen winter (WAG-W). There was no significant genotype × flush interaction for the two locations in Kenya so that for subsequent analyses they were treated as just two environments WIN and AGR. So in total, four different environments were identified and used for further studies.

Within our population in WAG-S, three traits were measured before bending; this stage is also referred to as the juvenile period. These traits were height (H), vigour (V) and the length of time before bending (BT). These traits exhibited transgressive segregation and had high heritabilities (0.73-0.82; Table 1).

**Table 1:** **Descriptive statistics of the morphological traits of the parents (4 replicates per parent) and the K5 population; BT days to bending (days from May 11), H plant height (cm) and V plant vigour (ranked 1–5), SL stem length (cm), SW stem width (mm), PS prickles on the stem, PP prickles on the petioles, NP number of petals, SW stem width (mm), CHL chlorophyll content, and SS number of side shoots**

**Environment**	**Trait**	**Parental**		**F1 progeny**			**All Environments**
		**P540**	**P867**	**K5 population**			
		**Mean ± SD**	**Mean ± SD**	**Mean ± SD**	**Range**	**H** ^**2**^	**H** ^**2**^
WAG-S	BT	1.50 ± 0.58	2.00 ± 0.00	2.07 ± 0.86	0.86-2.07	0.80	
WAG-S	H	27.75 ± 5.12	29.25 ± 2.50	27.04 ± 8.73	8.73-27.04	0.82	
WAG-S	V	3.00 ± 0.00	3.00 ± 0.00	2.98 ± 0.76	0.76-2.98	0.73	
AGR	**CHL**	61.90 ± 5.50	59.52 ± 3.78	58.46 ± 4.67	40.20-75.10	0.75	0.64
WAG-W	CHL	52.55 ± 4.63	48.31 ± 3.20	51.52 ± 5.01	33.10-68.50	0.68	
WIN	CHL	52.58 ± 2.50	53.81 ± 1.09	53.22 ± 3.36	41.10-62.55	0.72	
AGR	**NP**	34.00 ± 5.66	36.75 ± 2.61	33.97 ± 15.09	11-101	0.97	0.96
WAG-S	NP	30.00 ± 7.07	29.75 ± 3.59	31.67 ± 13.97	11-80	0.88	
WAG-W	NP	39.00 ± 5.66	41.38 ± 4.39	35.93 ± 15.77	13-113	0.90	
WIN	NP	36.97 ± 1.36	34.36 ± 1.01	34.52 ± 14.62	13-98	0.99	
AGR	**PP**	1.78 ± 0.51	0.38 ± 0.48	0.72 ± 0.52	0.00-3.00	0.93	0.89
WAG-S	PP	2.08 ± 0.42	0.50 ± 0.58	0.94 ± 0.59	0.00-3.33	0.89	
WAG-W	PP	1.83 ± 0.73	0.46 ± 0.37	0.74 ± 0.44	0.00-2.33	0.86	
WIN	PP	2.05 ± 0.27	1.00 ± 1.15	0.62 ± 0.43	0.00-2.15	0.87	
AGR	**PS**	10.67 ± 2.08	0.44 ± 0.62	7.76 ± 3.65	0.00-21.00	0.91	0.91
WAG-S	PS	8.00 ± 2.16	0.00 ± 0.00	7.17 ± 3.85	0.00-30.00	0.90	
WAG-W	PS	6.00 ± 1.80	0.00 ± 0.00	6.48 ± 3.45	0.00-19.5	0.89	
WIN	PS	13.06 ± 1.33	0.29 ± 0.46	7.62 ± 3.39	0.00-20.00	0.92	
AGR	**SL**	66.83 ± 4.65	75.62 ± 7.23	65.21 ± 9.41	42.00-101.00	0.84	0.84
WAG-S	SL	76.59 ± 9.22	82.50 ± 6.60	73.74 ± 11.49	39.00-111.00	0.86	
WAG-W	SL	90.08 ± 9.71	105.12 ± 7.69	94.42 ± 16.17	49-142.5	0.88	
WIN	SL	74.38 ± 4.46	73.78 ± 4.18	66.98 ± 8.80	39.50-101.00	0.91	
AGR	**SS**	1.00 ± 1.73	3.25 ± 1.79	2.07 ± 1.14	0.00-6.00	0.66	0.40
WAG-W	SS	1.42 ± 1.18	4.50 ± 0.71	3.31 ± 1.74	0.00-10.00	0.63	
WIN	SS	2.56 ± 0.40	3.40 ± 0.34	2.15 ± 1.05	0.00-8.00	0.74	
AGR	**SW**	7.84 ± 0.58	6.61 ± 0.83	6.43 ± 0.89	4.03-10.05	0.75	0.57
WAG-W	SW	6.14 ± 0.84	7.03 ± 0.83	6.75 ± 1.12	4.11-11.49	0.60	
WIN	SW	7.92 ± 0.65	6.93 ± 0.48	6.66 ± 0.69	4.65-10.00	0.77	

As can be seen in Table 1, the traits chlorophyll (CHL), number of petals (NP), stem length (SL), side shoots (SS) and stem width (SW) had ranges beyond those of the parents indicating transgressive segregation. For the traits prickles on stem (PS) and prickles on petioles (PP) the progeny had ranges exceeding the parents in one direction as one of the parents, P867, did not have prickles on the stem or petioles.

Significant differences between the performances of all the genotypes in each environment were found for all traits (*p < 0.001*). We also compared the performance of the genotypes across the three to four environments and we found significant differences in the performance of the genotypes means for the traits SL, SS, SW and CHL (*p < 0.001*). The mean SL in WAG-W was 94 cm, while in WAG-S, WIN and AGR the means were 74, 67 and 65 cm respectively (Table 1). The mean number of SS in WAG-W was 3.3 and in WIN and AGR 2.2 and 2.1, respectively. The mean SW in WAG was 6.8 mm and in WIN and AGR 6.7 and 6.4 mm. The average CHL ranged from 51.5 in WAG-W to 58.5 in AGR. The biggest difference in chlorophyll content range between the genotypes was in WAG-W with 35.4 and 34.9 in AGR. The lowest difference was in WIN with 21.5.

The mean of the number of petals in WIN, AGR and WAG-W were comparable at 34.5 to 35.9 but the average petal number dropped to 31.7 in WAG-S. The difference in the number of petals between the environments can be observed especially in the range of number of petals. In WAG-S the number of petals ranges from 11 to 80 whilst WAG-W the number of petals range was 13 to 113. In AGR the number of petals ranges from 11 to 101 petals. The mean number of petals ranged from 11 to 113 within the progeny, whilst the parents P540 and P867 had an average of 35 and 36 petals respectively. Both the parents and the K5 population would be classified as having double flowers.

The average numbers of prickles on the stem and petioles of P540 were 9.4 and 1.9 respectively. The average number of prickles on stems and petioles of P867 were 0.18 and 0.59 respectively. P867 stems were not completely devoid of prickles as expected at the beginning of the experiment. The range in the progeny did not seem to transgress beyond P540 for both prickle traits.

### Heritabilities

Heritability estimates among the traits ranged from 0.60 for SW in WAG-W to 0.99 for number of petals in WIN (Table 1). Across all the environments the heritability estimates ranged from 0.40 for SS and 0.96 for NP. The juvenile traits of BT, H and V had heritabilities of 0.80, 0.82 and 0.73 respectively. NP had the highest observed heritabilities in all the environments with a range of 0.88 to 0.99. The traits SL, PS and PP also had high broad sense heritabilities with a range from 0.84 to 0.93. These high heritabilities make a good basis for further genetic analysis. The traits CHL, SS and SW had lower heritabilities in each environment compared to the other traits with a range from 0.60 to 0.77. Across all the environments CHL, SS and SW still had the lowest heritabilities.

### Correlation among traits

Pearson correlation coefficients were calculated between the juvenile traits BT, H and V against all the adult phase traits in the four environments to give a measure of the strength of linear association (Table 2). Pearson correlation coefficients were also computed between the traits NP, PP, PS and SL in four environments and CHL, SS, SW in three environments (Table 3). Finally correlations were calculated between all the traits measured in each environment [see Additional file 2].

**Table 2:** **Pearson correlation coefficients with the juvenile traits; H plant height (cm), BT days to bending (days from May 11) and V plant vigour (ranked 1–5) against all the traits across the four environments of WAG-S, WAG-W, WIN and AGR**

	**BT**	**H**	**V**
BT_WAG_S			
H_WAG_S	−0.71**		
V_WAG_S	−0.55**	0.81**	
CHL_AGR	0.01	0.1	0.07
CHL_WAG_W	−0.09	0.13	0.13
CHL_WIN	0.01	0.22	0.32**
NP_AGR	0.09	−0.2	−0.19
NP_WAG_S	0.19	−0.19	−0.24*
NP_WAG_W	0.16	−0.2	−0.21
NP_WIN	0.09	−0.15	−0.19
PP_AGR	−0.07	0.08	0.14
PP_WAG_S	−0.05	0.05	0.14
PP_WAG_W	−0.01	0.07	0.13
PP_WIN	−0.06	0.08	0.13
PS_AGR	−0.37**	0.3*	0.16
PS_WAG_S	−0.34**	0.17	0.07
PS_WAG_W	−0.28*	0.27*	0.17
PS_WIN	−0.34**	0.24	0.08
SL_AGR	−0.46**	0.4**	0.22
SL_WAG_S	−0.39**	0.39**	0.21
SL_WAG_W	−0.45**	0.47**	0.28*
SL_WIN	−0.44**	0.43**	0.2
SS_AGR	−0.01	0.06	0.04
SS_WAG_W	−0.18	0.24*	0.31**
SS_WIN	0.07	0.00	0.16
SW_AGR	−0.26*	0.24	0.17
SW_WAG_W	−0.33**	0.33**	0.3*
SW_WIN	−0.26*	0.26*	0.2

These traits include SL stem length (cm), SW stem width (mm), PS prickles on the stem, PP prickles on the petioles, NP number of petals, SW stem width (mm), CHL chlorophyll content, and SS side shoots.**Correlation is significant at α = 0.001 *Correlation is significant at α = 0.01.

**Table 3:** **Pearson correlation coefficients of traits measured across environments**

**Trait**	**WAG-S.WAG-W**	**WAG-S.WIN**	**WAG-S.AGR**	**WAG-W.WIN**	**WAG-W.AGR**	**WIN.AGR**
**CHL**				0.29**	0.54**	0.33**
**NP**	0.93**	0.91**	0.88**	0.90**	0.88**	0.92**
**PP**	0.84**	0.73**	0.73**	0.66**	0.69**	0.75**
**PS**	0.84**	0.77**	0.70**	0.78**	0.71**	0.76**
**SL**	0.71**	0.71**	0.55**	0.65**	0.62**	0.69**
**SW**				0.32**	0.32**	0.29*

**Correlation is significant at α = 0.001 *Correlation is significant at α = 0.01. SL stem length (cm), SW stem width (mm), PS prickles on the stem, PP prickles on the petioles, NP number of petals, SW stem width (mm) and CHL chlorophyll content.

The trait BT which were the number of days between planting and bending of the branches was negatively correlated to the traits H and V in WAG-S (r = 0.71 and r = 0.55 respectively), PS in AGR (r = 0.37), PS in WAG-S (r = 0.34), PS in WIN (r = 0.34) and SW in WAG-W (r = 0.33). BT was also negatively correlated to SL in all the environments with AGR(r = 0.46), WAG-S (r = 0.39), WAG-W (r = 0.45) and WIN (r = 0.44). All the correlations were statistically significant (*p < 0.001*). Correlations between H and V in WAG-S (0.81), SW in WAG-W (r = 0.33) and SL in AGR, WAG-S, WAG-W and WIN (r = 0.40, r = 0.39, r = 0.47 and r = 0.43) were statistically significant (*p < 0.001*). The trait Vigour (V) was statistically significantly (*p < 0.001*) correlated to the traits CHL in WIN (r = 0.32) and SS in WAG-W (r = 0.31) (Table 2).

The correlation of the same trait between two environments was significant for all pairs of environments at *p* < 0.001 for the traits NP, SL, PS, PP, CHL and SW with one exception. The highest significant correlation was for the trait (NP) with a range of r = 0.88 to r = 0.93. The trait SL had significant correlations with a range or r = 0.55 to r = 0.71. The traits PS and PP also had high correlations across environments with a range of r = 0.70 to r = 0.84 for PS and r = 0.66 to r = 0.84 for PP. SW had low but significant positive correlations across environments with r = 0.29 to r = 0.32. CHL had a moderate correlation between the environments WAG-W and AGR, r = 0.54, but correlations were low for CHL for the environments WAG-W and WIN with r = 0.29 and WIN and AGR with r = 0.33. The correlation for SW for the environment pair WIN and AGR was only significant at *p* < 0.01 where r = 0.29 (Table 3). The trait SS had no significant correlations across any of the environments.

We were able to observe the correlation of all the traits within their environments [see Additional file 2]. All the correlations mentioned were statistically significant (*p < 0.001*). In WAG-S the trait H had a positive correlation to SL (r = 0.39) and V (r = 0.82). The traits PS was positively correlated to SL (r = 0.48). BT was negatively correlated to H (r = 0.69), PS (r = 0.31), SL (r = 0.31) and V (r = 0.58). In WAG-W there was a positive correlation between the traits PS and SL (r = 0.37), PS and SW (r = 0.31), SL and SS (r = 0.48), SL and SW (r = 0.66) and SS and SW (r = 0.65). The trait SL was negatively correlated to NP (r = 0.28). In WIN there was a positive correlation between PS and SL (r = 0.36) and SW (r = 0.41). The trait SL also had a positive correlation to SW (r = 0.61). In AGR the traits PP and PS were positively correlated (r = 0.35). The trait SW was also positively correlated to PS and SL and SS with r = 0.43, r = 0.63 and r = 0.30 respectively. The trait PS was positively correlated to SL (r = 0.43).

### Multivariate analyses

**Figure 2:**
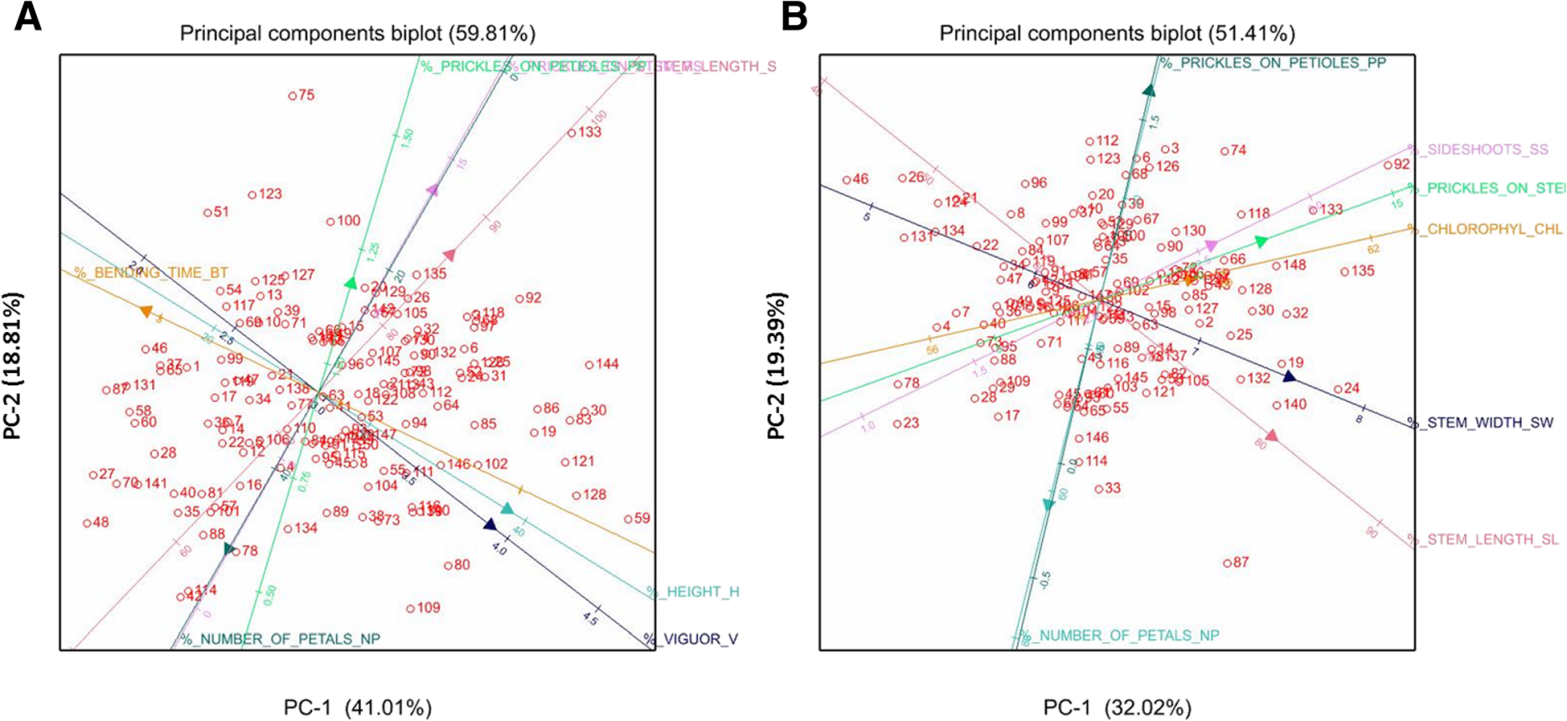
**Principal components biplots for all the traits, panel A for environment WAG-S; panel B for AGR.** The cosine of the angle between the lines approximates the correlation between the traits they represent. Arrows pointing in opposite directions mean negative correlations. Text explaining the traits might overlap due to the Genstat programme used.

Traits measured on the K5 population in the four environments WAG-S, WAG-W, WIN and AGR are shown in a PCA biplot (Figures 2A&B and Additional file 3). In WAG-S the first principal component accounted for 41% of the variation and 19% was explained by the second. For the environments WAG-W, WIN and AGR the first principal component accounted for 37%, 29% and 32% respectively and the second for 18%, 19% and 19%, respectively. There were differences in trait association across the four environments showing that the traits were partially influenced by the environment.

In WAG-S (Figure 2A) the traits H and V show an opposite direction from BT in agreement with a negative correlation coefficient. The traits PP, PS and SL have an opposite direction from NP, also indicating a negative correlation.

In WAG-W (Additional file 3) the traits SW, SL, SS and PS positively correlated together. The traits CHL correlates opposite from NP showing a negative association. There was no correlation between PP and the traits SW, SL, SS and PS.

In WIN (Additional file 3) the traits CHL, SS, PS, SW and SL were loosely correlated. The trait PP was positioned opposite from NP showing a negative association. There was no correlation between the traits NP and PP with the other traits.

In AGR (Figure 2B) the traits SS PS, CHL, were closely correlated. The trait PP was positioned opposite NP showing a negative association. SW and SL were also loosely correlated and were positioned opposite from NP showing a negative association.

### Genotype by environment interaction

For the traits chlorophyll content (CHL), number of petals (NP), prickles on petioles (PP), prickles on stem (PS), and stem length (SL) the genetic variance component was larger than the G × E interaction variance component. For the traits stem width (SW) and side shoots (SS) the G × E component was higher than the genetic variance component (Table 4). The traits that had a low ratio of σ^2^_ge_/σ^2^_g_ also showed a high value for heritability. For the traits NP, PP, PS the interaction is minimal. For the traits CHL, SW and SS we observed a much higher interaction ratio of 0.83, 1.43 and 3.13 respectively. The trait number of petals had the highest heritability of 0.96 and the lowest σ^2^_ge_/σ^2^_g_ ratio (0.08). The trait number of side shoots (SS) with the lowest heritability (0.40) also had the highest σ^2^_ge_/σ^2^_g_ ratio of 3.13.

**Table 4:** Estimates of variance components for genotypic variance and variance for genotype*environment interaction and ratio of genotype*environment interaction variance to genetic variance for all the morphological traits.

		**Source of variance**		
**Trait**	**Locations**	**Genotype σ** ^**2**^ **g**	**Genotype × Environment σ** ^**2**^ **ge**	**σ** ^***2***^ **ge/σ** ^**2**^ **g**
NP	4	272.47	21.13	0.08
PP	4	0.21	0.05	0.25
PS	4	11.70	2.65	0.23
SL	4	83.99	45.26	0.54
CHL	3	7.13	5.91	0.83
SS	3	0.23	0.72	3.13
SW	3	0.19	0.28	1.43

SL stem length (cm), SW stem width (mm), PS prickles on the stem, PP prickles on the petioles, NP number of petals, SW stem width (mm), CHL chlorophyll content, and SS side shoots.

The G × E interactions were further explored through a GGE biplot analysis. The first two principal components of the GGE biplots explained 97.52% (PC1 = 95.04% and PC2 = 2.47%) of the totals GGE variation for NP (Table 5). The summed explained variances of the first two principal components for the traits are listed in Table 5.

**Table 5:** **Trait-wise principal component 1 and 2 variance (PC1 and PC2) of total GGE variation in the traits NP, PP, PS, SL, CHL, SS and SW evaluated over four environments**

	**GGE**		
Trait	PC1	PC2	Sum
NP	95.04	2.47	97.52
PP	83.62	8.58	92.2
PS	84.39	6.77	91.16
SL	80.56	10.01	90.57
CHL	72.31	16.7	89.01
SS	66.67	20.02	86.69
SW	66.08	22.11	88.19

SL stem length (cm), SW stem width (mm), PS prickles on the stem, PP prickles on the petioles, NP number of petals, SW stem width (mm), CHL chlorophyll content, and SS side shoots.

**Figure 3:**
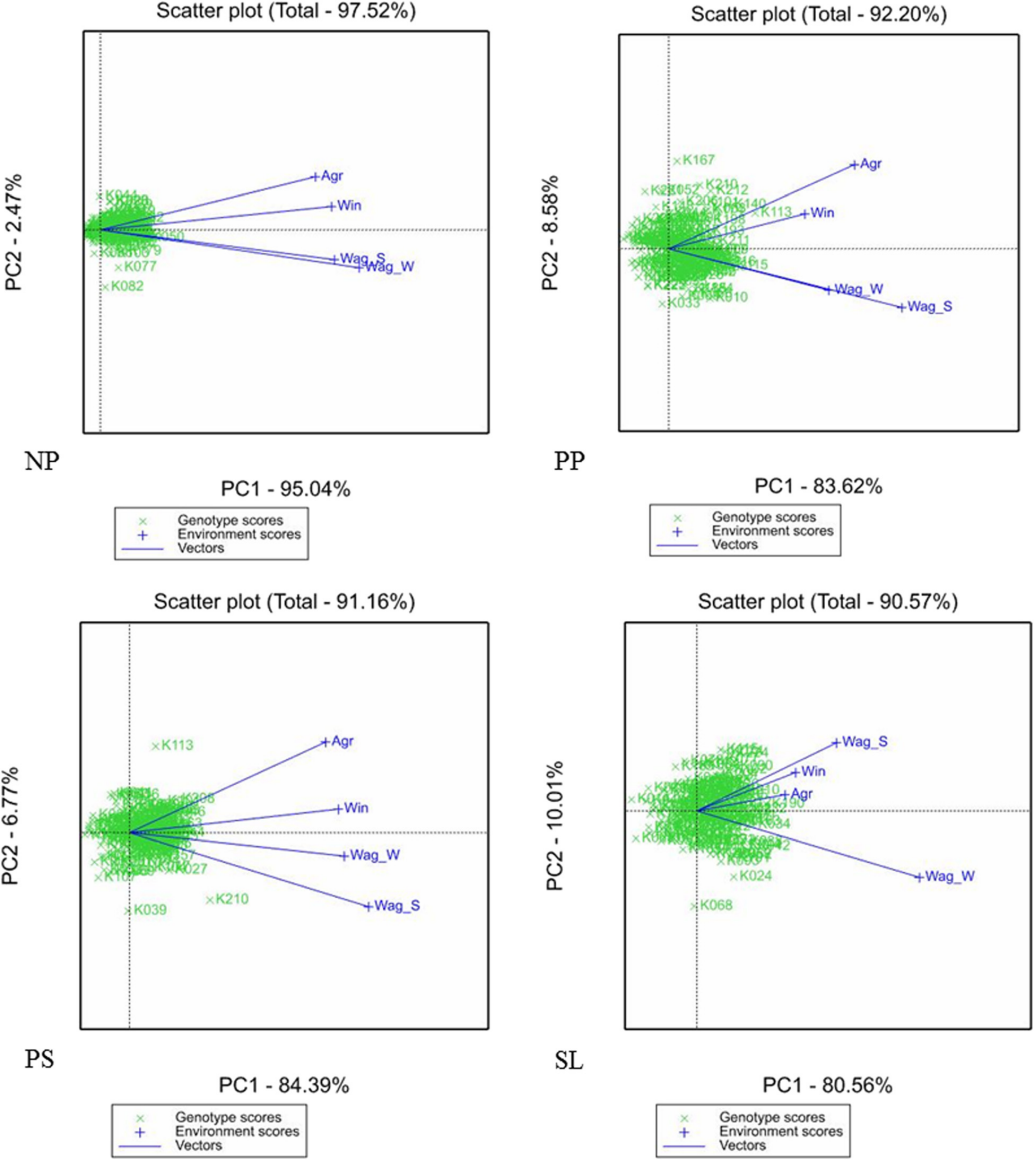
**GGE biplots for the morphological traits showing the relationship among the environments for NP (number of petals), PP (prickles on petioles), PS (prickles on stems) and SL (stem length).**

The summary of the interrelationships among the environments is presented in Figure 3 for NP, PP, PS and SL and in Additional file 4 for SS, SW and CHL. The environment vectors drawn from the biplot origin to connect the environments revealed positive PC1 scores for all the environments. There are sharp angles between all the four environments in this study indicating positive correlations amongst them. These results were confirmed by the Pearson correlations [see Additional file 2]. The plots show that for most of the genotypes the ranking was similar within the different environments. In all the environments the traits NP, PP and PS had low PC2 scores showing that the environments did not discriminate the genotypes. This result can be corroborated by the low ratios of σ^2^_ge_/σ^2^_g_ (Table 5). For the trait SL, the environment WAG-W was far from WAG-S (Figure 3), WIN and AGR. This meant that WAG-W discriminates the genotypes differently than the other three environments.

The length of the environment vectors from the biplot origin to the environment marker indicate how discriminating the environment is with respect to of the genotypes, with longer vectors being more discriminating [14]. For the trait NP the vectors were similar in length and had high PC1 scores. For the trait PS WAG-S and WAG-W were similar in discriminating genotypes. For the traits SS and SW the length of the environmental vector for WAG-W was longer than that of WIN and AGR thus the environment WAG-W was more capable of discriminating the genotypes.

The distance between two environments measures their similarity or dissimilarity in discriminating the genotypes [15]. For the trait NP, the environments WAG-S and WAG-W were similar in discriminating genotypes. Also, AGR and WIN were similar in discriminating genotypes. This is confirmed by the high positive correlations where WAG-S and WAG-W had (r = 0.93) and AGR and WIN (r = 0.92) as shown in Table 3.

## Discussion

The K5 population showed continuous variation for the studied traits. The transgressive segregation in both directions for most of the traits may suggest the involvement of multiple genes. The significant genotypic differences observed for all the traits showed there was sufficient genetic variation in the genotypes within the segregating population for future QTL (Quantitative Trait Loci) studies.

### Juvenile traits in relation to all other traits

Most breeders sow their seeds in the Netherlands under controlled glass greenhouse followed by selection under these conditions. Seedlings are selected under controlled greenhouse conditions in the first clonal selection. After further observation cuttings are made from the selected genotypes and they are sent to the tropics, to be grown in soil under plastic green houses. It was important for us to determine if the phenotype observed during the juvenile phase in the Netherlands could be used as an indicator of the performance of the genotypes in the tropics.

Within the K5 population we observed that there was a significant strong negative correlation between the length of the juvenile period (BT) and the final stem lengths (SL) of the genotypes, i.e. those genotypes that had a shorter juvenile period produced longer stems during production. This is in agreement with [16] who found a highly significant negative correlation between the juvenile period and plant length. Although the BT also had a significant negative correlation to vigour (V), there was no significant correlation between V and SL. This is because plant vigour is based not only on the height of the plant but the number of stems, number of leaves and the branching present at time of scoring. A seedling is therefore only bent once it has achieved a certain level of vigour. This explains why we have a strong significant correlation between the traits BT and V. The time from seedling emergence to flowering can therefore be used to predict stem length, but not vigour as a number of other traits other than plant height are incorporated into the trait V. It is important to take note that depending on the population the juvenile period will vary. It was observed by [17] that there is considerable genetic variation between rose populations in the number of days from germination to first flower and accompanying shoot lengths but these traits are correlated within each population. The end of the juvenile period is determined by the appearance of the flower bud. At this point most commercial breeding programs typically rough out 75-95% of seedling at first bloom. Strong emphasis and early selection for floral traits leads to the possibility that population sizes may be so strongly reduced that there may be little variation left to make strong gain from selection for non-floral traits which take more time to express themselves [7]. This results in a limitation on the genetic diversity of future breeding material. Our results and those of [17] prove that in addition to flower colour, measuring the days to bud appearance are good indicators of the stem length for the selected roses.

### Number of petals

The high observed broad sense heritabilities for number of petals were an indicator that this trait has a strong genetic component with not much environmental influence. Heritability for number of petals was found to be high in our tetraploid population similar to what was observed in the diploid populations analysed by [18]. The analysis of variance components showed that NP had a considerably higher genetic variance component than the G × E component, the σ^2^_ge_/σ^2^_g_ ratio (0.08) illustrating that there is only a very small contribution of the interaction. The observed difference in the number of petals across the environment can be found in the ranges where WAG-S had a lower average number of petals compared to WAG-W, WIN and AGR. This was also corroborated by the GGE biplot which showed the environments did not discriminate the genotypes. The presence of petal numbers lower and higher than the parents is an indication of transgressive segregation. This would suggest that the observed variation within the double flowers is controlled by multiple genes. On the other hand, a single dominant locus responsible for the switch from single flower to double flower phenotype has been identified on LG3 in the integrated consensus map (ICM) [6,8-10]. In the near future a QTL analysis will be carried out to determine if the QTLs responsible for the variation in the number of petals of double flowers co-localise with the previously identified *Blo* QTL for the single to double flowers switch.

Within the K5 population there were significant differences (*p < 0.001*) in number of petals among the genotypes. This was first observed by [19] who showed that in a self-pollinated progeny of the tetraploid cultivar Golden Sceptre there was a wide distribution of petal numbers suggesting that the varying petal number among double flowers is due to multiple genes. So, the variation in petal number among the double flowered individuals also seems to have a heritable component. This has been further confirmed by [8], who illustrated that petal number in double flowers was variable and could be scored quantitatively to show genetic variability.

We also observed a difference in the number of petals across the environment for the same genotypes. Within our population, we found that in WAG-W we had the highest average number of petals and the lowest number of petals in WAG-S. It has been reported that at higher temperatures there is a decrease in the petal number [20] and at low temperatures there is an increase in the number of petals [21]. A study in the size and weight of buds showed an increase in winter and the difference observed did not come from an increase in petal size but from an increase in the number of petals [22]. Taking into consideration the greenhouse environment we can conclude that in the K5 population it wasn’t just the higher temperature that resulted in a decrease in petal numbers but also the differences in temperature. WAG-S which had the lowest number of petals had the largest difference in day and night temperature of 24°C whilst WAG-W which the largest number of petals had the lowest difference in temperature of 6°C. Taking into consideration the observations of [20] and [22] that a higher flower weight is the result of an increase in petal number, the finding of [23] that in his cultivars, the average flower weight was higher when the day and night temperatures were the same than when the day temperatures were higher than the night temperatures confirms our results. This might also explain why the same genotypes grown in WAG-W which had the lowest difference between the day and night temperatures of 6°C had the highest number of petals at 13–113. WAG-S which had the largest difference between the day and night temperatures of 24.1°C had the lowest number of petals 11–80.

### Prickles on stem and petioles

Within the K5 population in all the environments we observed that the prickles on petioles and the prickles on stem exhibited transgressive segregation. This indicates that multiple genes may be responsible for this trait. The transgressive segregation was unidirectional as the parent P867 did not have prickles on stem or on petioles. Both of these traits had high heritabilities and low genotype by environment interaction. The traits PS and PP had low PC2 percentages of 8.58% and 6.77%. Between PS and PP there was a weak but significant positive correlation (*p < 0.001*) in only one environment AGR (r = 0.35). In the other environments the two traits did not have any significant correlation. In the PCA plots in three of the environments PP and PS did not show any strong positive or negative correlation. The low correlation, in combination with the high heritabilities, is a good indicator that the two traits segregate independently. It was observed in other populations that the absence of prickles on the stem and of prickles on the petiole indeed segregate independently [24]. These authors suggested that the absence of prickles on petioles is controlled by a single recessive gene. To our surprise, the one parent that was classified as prickle free for the stems still produced a couple of prickles after some years in the greenhouse, this could be a result of the plant ageing as we never found the prickles in the seedlings. Nobbs [25] and Bayse [26] observed within their populations that some of their cultivars classified as prickle free also produced prickles after some years. Druitt and Shoup [7] and Bayse [25] concluded that conditions like temperature shock, freezing, change in soil make up caused putative thornless rose to sprout thorns. They also concluded that many thornless roses are chimeras, consisting of mutant thornless tissue that grows together with normal thorny tissue.

In the environment WAG-W the trait PS had intermediate positive correlations to the traits SL and SW, 0.37 and 0.31 respectively. This data shows that longer or wider stems do not automatically translate to more prickles on the stem. Further analysis will be performed to determine whether there is a possible genetic linkage between alleles influencing these traits.

### Stem length

The trait SL had consistently high heritabilities ranging from 0.84 to 0.91 which indicated that this trait would be suitable for further genetic studies. Broad sense heritability at the genotypic mean level is a reliable indicator for the possibilities of selecting for genetic characteristics in a cross [27]. Within the K5 population the stem length was significantly higher in winter (WAG-W) than in summer (WAG-S) and both Kenyan locations (WIN and AGR). The analysis of variance components showed that for SL the genetic variance component was larger than the G × E component, the σ^2^_ge_/σ^2^_g_ ratio was 0.54, illustrating that there is an interaction present. The GGE biplot illustrates that genotypes responded differently in WAG-W compared to WAG-S, WIN and AGR.

The greenhouse in WAG-W had on average a lower temperature than the other environments and also the lowest difference between maximum and minimum temperature. It also had a much higher relative humidity. The observed difference in performance of the genotypes across the environments can be attributed to the varying climatic conditions in the greenhouse. This is in agreement with [20] who observed that the length of the flowering stem was significantly reduced with increasing temperature. This can be explained by the fact that stem length at a certain developmental stage depends on both rate of growth and the rate of development. When the rate of development is promoted more than the rate of growth at higher temperatures, plant length is reduced [28,29]. It has been suggested that at higher temperature the rate of development is accelerated thus the plant reaches the developmental stage for florogenesis and anthesis much earlier. At anthesis, auxin production is stopped resulting in shorter stems [20].

### Stem width

The low (but still significant) correlations of SW across environments are in agreement with the high observed σ^2^_ge_/σ^2^_g_ ratio of 1.43. The GGE biplot shows that SW responded differently to different environments. In WIN and AGR the trait SW had a similar response whilst WAG-W discriminated the genotypes differently. As the traits were positively correlated there was no crossover interaction observed. We also observed that SW showed significant but low positive correlations to the traits SL, SS and PS [see Additional file 2]. Further genetic studies would enable us to identify if these traits are genetically linked.

### Number of side shoots

\The trait SS did not have high positive or negative correlations across any of the environments. This trait had the lowest heritability of the traits studied and we observed a very high genotype × environment interaction. The GGE plot shows that the genotypes responded differently for this trait in different environments. The G × E variance component for this trait was much higher than the genotypic variance component thus we can conclude the environment strongly affects this trait.

### Chlorophyll

The trait Chlorophyll also exhibited transgressive segregation and had high heritabilities ranging from 0.68 to 0.75 across the environments. This trait also had strong positive correlations across all the environments. This trait showed a high interaction when its genotypic component was compared to the G × E component. This was also observed on the GGE biplot which showed that WAG-W discriminated the genotypes differently for this trait from AGR and WIN. This trait was shown to have a non cross-over G × E interaction. As there was no correlation between this trait and stem length, stem width or vigour trait we can conclude that chlorophyll content is genetically independent of this trait and therefore cannot be used to predict the vigour of a plant.

## Conclusions

In the last decade rose breeding has had to rapidly adapt to the change in growing climate as production locations have shifted from predominantly seasonal European climate to more constantly warm climates in the tropics. This meant that more trials had to be done to identify if the European selected varieties were suitable for the warmer environments. We choose to study the effects of environment on the traits NP, PS, PP, SL, SW, SS and CHL as these are the traits that form the basic plant structure. If a breeder was able to understand how these traits are inherited and how they respond to the different environments they would be able to make a more structured breeding program. Currently an ideal rose plant would have at least 30 petals, no prickles on the stem or petioles, at least 50 cm stem length and very few side shoots. The stem width is relative to the stem length.

The traits had a high heritability thus enabling the breeder to actively breed for or against these traits. It was also important to understand how the environment would affect these traits. We can now conclude that in colder climates you get longer stems but the traits prickles on stem and petals are not affected by the environment. For the number of petals we saw that to increase the number of petals we need to reduce the difference in day and night temperatures. The lack of strong positive correlations to chlorophyll is an indicator that the darker green leaves is aesthetically more pleasing but that does not translate to a more vigorous plant.

Results attained by this experiment showed that we have different magnitudes of non-crossover G × E interactions. For the traits NP, PS and PP with a low interaction and high heritability, selection can be done at any of the environments. Thus these traits can be confirmed at the breeding site. For the traits SL, SW, SS and CHL that had a higher interaction selection for or against these traits should be done at or at least verified at the production location.

## Additional files

Additional file 1:
**Temperature and relative humidity of the greenhouse during the experiments.**
Additional file 2:
**Pearson correlation coefficients of traits per environment in WAG-S, WAG-W, WIN and AGR.**
Additional file 3:
**Principal components biplots for all the traits, in the environments WAG-W (panel A) and WIN (panel B).**
Additional file 4:
**GGE biplots for the morphological traits showing the relationship among the environments for SS (side shoots), SW (stem width) and CHL (chlorophyll content).**
