# Supplementary material for: Genetic variation, heritability and genotype by environment interaction of morphological traits in a tetraploid rose population
Source: BMC Genet. 2014 Dec 20;15:146. doi: 10.1186/s12863-014-0146-z (PMC4293809; doi:10.1186/s12863-014-0146-z)
Supplement: Additional file 1: — Temperature and relative humidity of the greenhouse during the experiments. [file 12863_2014_146_MOESM1_ESM.docx]

**Additional file 1:** Temperature and relative humidity of the greenhouse during the experiments.

|  |  | **Relative humidity %** | | | |  | **Temperature (^o^C)** | | | |
| --- | --- | --- | --- | --- | --- | --- | --- | --- | --- | --- |
| **Location** | **Environment** | Mean | Min | Max | Max-Min |  | Mean | Min | Max | Max -Min |
| Netherlands | WAG-S | 82 | 42 | 100 | 58 |  | 21 | 11 | 36 | 24 |
| Netherlands | WAG-W | 95 | 79 | 100 | 21 |  | 19 | 16 | 22 | 6 |
| Kenya | WIN | 58 | 37 | 83 | 47 |  | 28 | 20 | 35 | 15 |
| Kenya | AGR | 49 | 25 | 75 | 50 |  | 25 | 20 | 34 | 14 |
