# Supplementary material for: Genetic variation, heritability and genotype by environment interaction of morphological traits in a tetraploid rose population
Source: BMC Genet. 2014 Dec 20;15:146. doi: 10.1186/s12863-014-0146-z (PMC4293809; doi:10.1186/s12863-014-0146-z)
Supplement: Additional file 2: — Pearson correlation coefficients of traits per environment in WAG-S, WAG-W, WIN and AGR. [file 12863_2014_146_MOESM2_ESM.docx]

**Additional file 2:** Pearson correlation coefficients of traits per environment in WAG-S, WAG-W, WIN and AGR. The traits include H plant height (cm), B days to bending (days from May 11),V plant vigour (ranked 1-5) SL stem length (cm), SW stem width (mm), PS number of prickles on the stem, PP number of prickles on the petioles, NP number of petals, SW stem width (mm), CHL chlorophyll content, and SS number of side shoots. **Correlation is significant at α=0.001, *Correlation is significant at α= 0.01

**WAG-S WAG-W**

| CHL |  |  |  |  |  |  |
| --- | --- | --- | --- | --- | --- | --- |
| NP | -0.20 |  |  |  |  |  |
| PP | 0.19 | -0.12 |  |  |  |  |
| PS | -0.07 | -0.19 | 0.11 |  |  |  |
| SL | 0.26* | -0.28** | -0.14 | 0.37** |  |  |
| SS | 0.28* | -0.20 | -0.07 | 0.11 | 0.48** |  |
| SW | 0.22* | -0.18 | -0.09 | 0.31** | 0.66** | 0.65** |
|  | CHL | NP | PP | PS | SL | SS |

| BT |  |  |  |  |  |  |
| --- | --- | --- | --- | --- | --- | --- |
| H | -0.69** |  |  |  |  |  |
| NP | 0.15 | -0.17 |  |  |  |  |
| PP | -0.03 | 0.03 | -0.08 |  |  |  |
| PS | -0.31** | 0.22 | -0.31** | 0.17 |  |  |
| SL | -0.31** | 0.39** | -0.27* | 0.05 | 0.48** |  |
| V | -0.58** | 0.82** | -0.21 | 0.1 | 0.13 | 0.22* |
|  | BT | H | NP | PP | PS | SL |

**WIN AGR**

| CHL |  |  |  |  |  |  |
| --- | --- | --- | --- | --- | --- | --- |
| NP | -0.12 |  |  |  |  |  |
| PP | 0.04 | -0.18 |  |  |  |  |
| PS | 0.07 | -0.16 | 0.35** |  |  |  |
| SL | 0.16 | 0.05 | -0.14 | 0.43** |  |  |
| SS | 0.18 | -0.26* | 0.03 | 0.11 | 0.19 |  |
| SW | 0.24* | 0.02 | 0.07 | 0.43** | 0.63** | 0.30** |
|  | CHL | NP | PP | PS | SL | SS |

| NP | -0.06 |  |  |  |  |  |
| --- | --- | --- | --- | --- | --- | --- |
| PP | 0.1 | -0.27* |  |  |  |  |
| PS | -0.1 | -0.11 | 0.23* |  |  |  |
| SL | 0.07 | 0.04 | -0.1 | 0.36** | |  |
| SS | 0.08 | -0.1 | 0.09 | 0.04 | 0.20* |  |
| SW | 0.19* | -0.01 | 0.07 | 0.41** | 0.61** | 0.20* |
|  | CHL | NP | PP | PS | SL | SS |
