# Supplementary material for: Genetic variation, heritability and genotype by environment interaction of morphological traits in a tetraploid rose population
Source: BMC Genet. 2014 Dec 20;15:146. doi: 10.1186/s12863-014-0146-z (PMC4293809; doi:10.1186/s12863-014-0146-z)

**Additional file 3:** Principal components biplots for all the traits, in the environments WAG-W (panel A) and WIN (panel B). The cosine of the angle between the lines approximates the correlation between the traits they represent. Arrows pointing in opposite directions mean negative correlations.


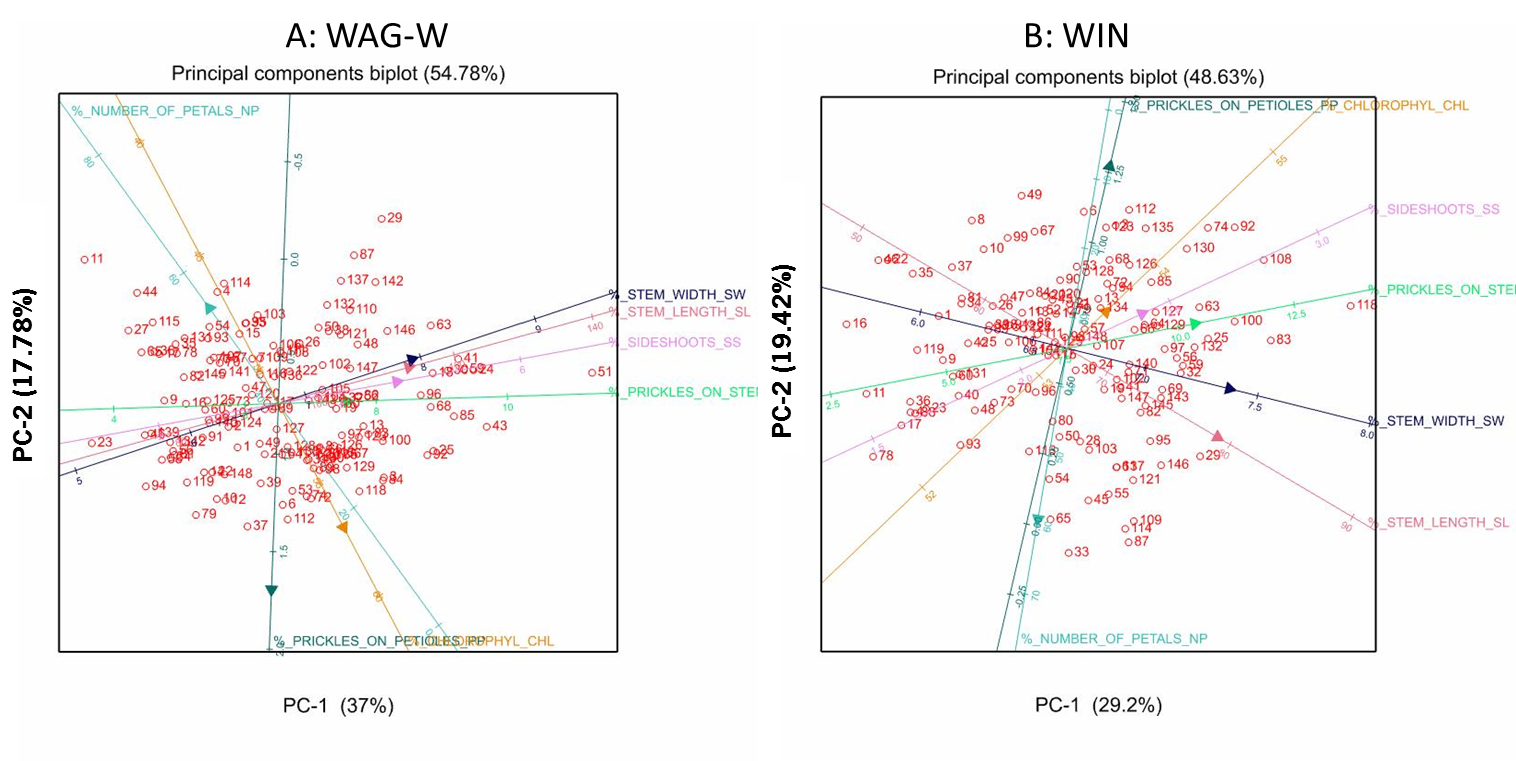

Supplement: Additional file 3: — Principal components biplots for all the traits, in the environments WAG-W (panel A) and WIN (panel B). [file 12863_2014_146_MOESM3_ESM.docx]
