# Supplementary material for: Genetic variation, heritability and genotype by environment interaction of morphological traits in a tetraploid rose population
Source: BMC Genet. 2014 Dec 20;15:146. doi: 10.1186/s12863-014-0146-z (PMC4293809; doi:10.1186/s12863-014-0146-z)

**Additional file 4:** GGE biplots for the morphological traits showing the relationship among the environments for SS, SW and CHL.


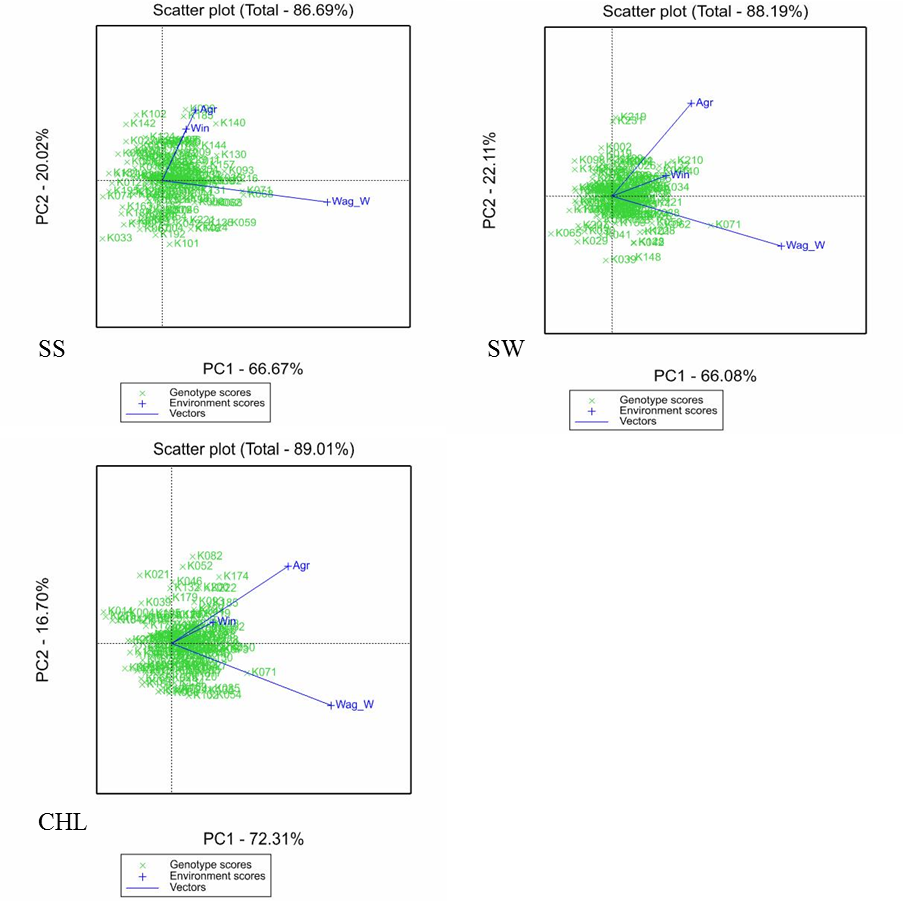

Supplement: Additional file 4: — GGE biplots for the morphological traits showing the relationship among the environments for SS (side shoots), SW (stem width) and CHL (chlorophyll content). [file 12863_2014_146_MOESM4_ESM.docx]
